# ANN deformation prediction model for deep foundation pit with considering the influence of rainfall

**DOI:** 10.1038/s41598-023-49579-z

**Published:** 2023-12-19

**Authors:** Xing Wei, Shitao Cheng, Rui Chen, Zijian Wang, Yanjun Li

**Affiliations:** 1 Department of Geotechnical Engineering, School of Civil Engineering, Southwest Jiaotong University, Chengdu, 610031 China; 2 Sichuan Vocational and Technical College of Communications, Chengdu, 611130 China

**Keywords:** Civil engineering, Mathematics and computing

## Abstract

Deep foundation pits involving complex soil–water-structure interactions are often at a high risk of failure under heavy rainfall. Predicted deformation is an important index for early risk warning. In the study, an ANN model is proposed based on the Wave Transform (WT), Copula method, Convolutional Neural Network (CNN) and Long Short-Term Memory Neural Network (LSTM). The total deformation was firstly decomposed into low and high frequency components with WT. The CNN and LSTM were then used for prediction of the two components with rolling training and prediction. The input variables of the CNN and LSTM were determined and optimized based on the correlations analysis of Copula method of the two components with different random variables, especially with the rainfall. And finally, the predicted total deformation was obtained by adding the two prediction components. A deep foundation pit in Chengdu, China was taken as a case study, of which the horizontal deformation curves at different measuring points shows three types of developed trend, as unstable, less stable, and stable types. The predictions of the deformations of different development types by the proposed ANN model show high accuracies with a few input variables and can accurately prompt risk warning in advance.

## Introduction

Deep excavations for construction of metro stations, building basements and other underground facilities have extensively emerged over recent years in the densely populated cities. Owing to the complexity and variability of subsurface conditions, deep foundation pits that involve complex soil–water-structure interactions are often at a high risk of failure related to heavy rainfall^1–3^. Rainfall often increases the soil unit weights, decrease the matric suction or increase the pore water pressure, decrease the strength of soils, and sometimes induces seepage erosion^4^. For some soil with unstable structures such as expansive soil or collapsible loess, cracks and settlement troughs may exist in the upper stratum and provide preferential pathways for water infiltration^4,5^. These variations in soil strata due to the rainfall would lead to internal stresses and horizontal deformation in the retaining structures. Therefore, the deformation of the retaining structures can reflect the current state of the stability of these deep foundation pits. During construction of deep foundation pits, the deformation of the foundation pits is usually monitored to ensure the safety of the foundation pits and surrounding structures^6^. The foundation pit might be judged as unstable when the prediction deformation exceeded the threshold or showed an unpredicted acceleration. However, because the deformation of soils and structures is usually nonlinear and influenced by multiple factors, it is hard to predict accurately^7^.

The commonly-used prediction methods of nonlinear deformation in geotechnical engineering can be grouped into two categories: the physical-based models and data-based models. For physical-based models, numerical simulation method is usually adopted to conduct an inverse analysis using the measured data to infer the current mechanical parameters of soils^6,8–12^. The reasonable soil parameters using in the numerical simulation is crucial for accurately predicting deformation, but the soil parameters measured in the laboratory and the actual soil parameters are often different^13^_._ Based on actual monitoring data, the inverse analysis method can obtain the actual soil parameters^6,14,15^. Some researchers took the excavation sequence into consideration^11,16–20^. To improve the calculation efficiency, the intelligent algorithms are introduced in the inverse analysis method^21–24^. However, physical-based models are still complex and time consuming, as they need to consider many conditions and some conditions cannot be directly obtained^25–27^. The data-based models can directly predict the deformation by the measuring deformation data, which are simple and efficient with high accuracy and only limited input data are required. Back Propagation Neural Network (BP), Support Vector Machine and Gray Verhulst model are developed to predict the deformation of foundations^28–30^, the deformation of slopes^31,32^, and the deformation of foundation pits^33–36^.

Among these models, BP model is the most widely used model, which is a feedforward multiple-layer perceptron model using error back-propagation learning algorithm^37–39^. A representative BP neural network consists of three layers for the structure: an input layer, a hidden layer, and an output layer^40^. The BP model has strong abilities of information processing, self-learning, nonlinear mapping, error feedback adjustment, and fault tolerance^37–41^. However, BP model also has some disadvantages such as slow convergence speed, long training time, difficult in achieve the overall optimum, and its prediction performance greatly depends on the random selection of initial weights and thresholds^35,42^. And the BP model requires large quantity training data and the data need to be widely representative^34,43^. For actual engineering, the requirement of the data could hardly be satisfied. Therefore, the BP model might be adequately trained due to limited training data, which may result large error in prediction^44^. Some researchers revised BP model with parameter optimization progress by new algorithms to avoid the local optimal solution and poor prediction accuracy. The revised BP models, such as the GA-BP, the PSO-BP and the SSA-BP models show high prediction accuracies in applications^45–47^. Some researchers adopted ANN with different structures, as the Convolutional Neural Network (CNN) and the Long Short-Term Memory (LSTM) neural network, to analyze the time series in the case of limited information. CNN is popular in computer vision and image processing, which uses convolution layers extract the spatial information in images, and with fully connected layers to store information in time-series data^48,49^. With convolution layers, the CNN model can effectively extracts the features of the time-series and deal with time series problem effectively^47–55^. One dimensional convolution CNN model is widely used in data series analyzing^47–52^, and the input can be one-dimensional^53,54^ or multi-dimensional arrays^55^. CNN may increase the accuracy up to 30% and train models twice faster than other algorithms such as RNN, GRU, and LSTM^56^. CNN weight division can reduce the number of parameters to increase the efficiency of model learning^54–57^. The CNN models can solve various time-series data, such as univariate, multivariate, multi-step, and multivariate multi-step model^56^. The LSTM network is a type of revised Recurrent neural network (RNN), which is a powerful and robust type of ANN that uses existing time-series data to predict the future data over a specified length of time^57^. The RNN model can only recollect the recent information^58^, and the main drawback of the RNN model is its shorter memory to remember the features, vanishing and exploding gradients^59,60^. To revise the RNN model, LSTM adopts memory blocks with three gate units as input, output, and forget gates, which perform the role of the normal neurons in the hidden layers^61^. These gates help in updating and controlling the flow of information through the memory blocks. The applications in predicting the market movement show that LSTM provides better prediction capabilities compared to random forest, and is very suitable for predicting volatility^62,63^. And the LSTM model is able to model problems with multiple input variables^64^.

The deformation of deep foundation pit evolving geo-material is complex nonlinear dynamic process and is influenced by different conditions. Therefore, decomposing the deformation into several components with different frequencies and predicting each component separately with different model based on their features might be a good approach. In this study, the deformation of foundation pit is decomposed into two components, low and high frequency components, by Wavelet Transform (WT). And then, based on the features of the two components, CNN and LSTM neural networks are adopted to predict the two components separately. In addition, the input variables of the CNN and LSTM neural networks are determined by analyzing the correlation of the two components with different random variables by Copula method. Consequently, an ANN deformation prediction model for foundation pit with consideration of the rainfall based on WT, CNN, LSTM and Copula method was proposed.

## Methods

### WT method and the decomposition of the deformation

In contrast to Fourier analysis, which decomposes the signal into a series of superposition of sinusoidal waves of different frequencies, WT method decomposes the signal into a series of superposition of wavelet functions. For a given signal *f*(t), discrete wavelet transform is generally used, and the signal decomposition can be expressed as,

1 $$f(t) = \sum\limits_{i,j = - \infty }^{\infty } {c_{i,j} \phi_{i,j} (t)}$$

where, $c_{i,j}$ is the discrete wavelet coefficient and $\phi_{i,j} (t)$ is the discrete wavelet function.

The method of approximating signals with irregular wavelet functions enables WT to have the ability to analyze local information in a superior manner in the time domain. SymN wavelets are a commonly used type of wavelet function, where N is the vanishing moment^65^. The low-frequency and high-frequency energy after WT analysis are related to the frequency characteristics of the signal and the vanishing moment N of the wavelet. The selection of decomposition layers also affects the decomposition effect. In the first layer of decomposition, the WT decomposes the original signal into a high frequency signal and a low-frequency signal, the second layer further decomposes the low frequency signal into a high-frequency signal and a low-frequency signal. Whether to continue decomposing the low-frequency signal depends on the given number of the decomposition layers. Finally reconstruct the decomposed signals to obtain the low frequency component and the high frequency component^66^.

For a foundation pit, the factors that affect the development of deformation curves can be divided into two groups, as long-term and short-term factors. Long-term factors include the excavation process, creep of support structures, and long-term rainfall; while short-term factors include the short-term disturbance factors such as the operation of construction vehicles and equipment, short-term rainfall and evaporation, earthquakes etc. Based on the different influence factors, the deformation of the foundation pit can be decomposed into two components with low and high frequencies using the WT, as

2 $$H({\text{t}}) = H_{{\text{L}}} ({\text{t}}) + H_{{\text{H}}} ({\text{t}})$$

where, *H* is the total horizontal deformation; t is time and the unit is day; *H*_L_ and *H*_H_ are the low frequency component and the high frequency component, respectively.

### Correlation analysis based on Copula method

Copula method can combine the empirical distribution function of two random variables to reflect the degree of correlation between the variables. Kernel density estimation is adopted to determine the empirical distribution function of a random variable. Let *X*1, *X*2, …, *X*n be independent random variables identically distributed as a random variable *X*. The kernel density estimation $f_{{\text{n}}} (x)$ at a point *x* is defined as^67^,

3 $$f_{{\text{n}}} (x) = \frac{1}{nh}\sum\limits_{i = 1}^{n} {k\left( {\frac{{x - X_{{\text{i}}} }}{h}} \right)}$$

where, *h* is the window width, and *n* is the sample size, *k*(*x*) is the kernel function and defined as^68^,

4 $$k(x) = \frac{1}{{\sqrt {2\pi } }}\exp \left( { - \frac{{x^{2} }}{2}} \right)$$

The function *f*_n_(*x*) is used to calculate the probability of a random variable *X* near a specific value *x*. When calculating the probability that the random variable *X* falls into the range as *x* less then *x*_p_, it is necessary to introduce the probability distribution function *F*(x < *x*_p_), which is the integral of the probability density function *f*_n_(*x*) over this range. Therefore, the empirical probability distribution function *F* is defined as,

5 $$F(x < x_{{\text{p}}} ) = \int_{ - \infty }^{{x_{{\text{p}}} }} {f_{{\text{n}}} (x)} {\text{d}}x$$

Assuming that the empirical distribution functions *F* and *G* of random variables *X* and *Y* were obtained through Eqs. (3) to (5). Then, a joint distribution function *C* for variables *X* and *Y* with empirical distribution functions *F* and *G* can be obtained by Frank-Copula function, as

6 $$C(F,G;\theta ) = - \frac{1}{\theta }\ln \left[ {1 + \frac{{(e^{ - \theta F} - 1)(e^{ - \theta G} - 1)}}{{e^{ - \theta } - 1}}} \right]$$

where, *θ* is the function parameter. And the Kendall Rank Correlation Coefficient *τ* is calculated as,

7 $$\tau = 1{ + }\frac{4}{\theta }\left[ {\frac{1}{\theta }\int\limits_{0}^{\theta } {\frac{x}{{e^{x} - 1}}{\text{d}}x} - 1} \right]$$

The larger the value of *τ*, the higher the correlation between the two variables *X* and *Y*.

The data of different variables which are easily accessible and can be fed into the ANN model fall into two categories: the deformation and the rainfall. The deformation data might be input to the ANN model are the data of the low and high components at time t, *H*_L_(t) and *H*_H_(t), deduced from measured deformation, while the rainfall data include daily rainfall at time t *R*(t) and the average rainfall *R*_m_(n, t), which is average rainfall for n days at time t and defined as,

8 $$R_{{\text{m}}} ({\text{t,n}}) = \frac{1}{{\text{n}}}\sum\limits_{{\text{i = t - n + 1}}}^{{\text{t}}} {R({\text{i}})}$$

where, *R*(i) is the daily rainfall at time i.

For the low frequency component *H*_L_, three groups of correlation analyses were conducted, as followings

1. The correlations between the items of the low frequency component time series.
  {*H*_L_(t), *H*_L_(t−1),…., *H*_L_(n+1)} and {*H*_L_(t−n), *H*_L_(t−n−1),…., *H*_L_(1)}
2. The correlation between the times series of incremental components and daily rainfall.
  {∆*H*_L_(t), ∆*H*_L_(t−1),…., ∆*H*_L_(n+1)} and {*R*(t−n+1), *R*(t−n),…., *R*(1)}
3. The correlation between the times series of incremental components and the average daily rainfall.
  {∆*H*_L_(t), ∆*H*_L_(t−1),…., ∆*H*_L_(1)} and {*R*_m_(t, n), *R*_m_ (t−1, n),…., *R*_m_ (1,n)}.

Noted that n is an integer greater than or equal to 1. The Copula method is used to analyze two random variables with same size and one coefficient *τ* is obtained for the two variables. Therefore, for the analysis of each group, n values of coefficients *τ* should be obtained. The average values of *τ* for the three groups would be compared firstly to determine which variable should be input. And then, the variation of the *τ* against n for one variable would be analyzed to determine how many data should be input for the variable.

For the high frequency component *H*_H_, two groups of correlation analysis were conducted, as

1. The correlation between the items of the high frequency component time series.
  {*H*_H_(t), *H*_H_(t−1), …, *H*_H_(n+1)} and {*H*_H_(t−n), *H*_H_(t−n−1), …, *H*_H_(1)}
2. The correlation between the times series of high frequency components and rainfall.
  {*H*_H_(t), *H*_H_(t−1),…., *H*_H_(n+1)} and {*R*(t−n+1), *R*(t−n),…., *R*(1)}

And then analyze the average values of *τ* for each group and the variation of *τ* against n in each group to determine the input data for the LSTM model.

### The CNN neural network for prediction of *H*_L_

The low frequency component reflects the increasing trend of the horizontal deformation of retaining structures during the construction of a foundation pit. Because the current data is a continuation of the previous state, there is a certain correlation between the items of the time series. The CNN with convolutional kernel is good at extracting the data features and the dependencies among the data, therefore CNN is adopted to predict the low frequency component. The architecture of a CNN network is usually composed of one input layer, several convolutional layers, several pooling layers, one fully connected layer, and one output layer, and the pooling layers are optional for CNN. More information can be retained without pooling layer which is helpful in improving the accuracy and robustness of the model. For the adopted CNN model, there are two convolutional layers, one fully connected layer, and no pooling layer.

Three variables, as *H*_L_, *R* and *R*_m_, are optional for the CNN model, which means three sequences are needed before optimizing. And the optional input data set for CNN model denotes as $V_{{{\text{CNN}}}}$, and is presented as,

9 $$V_{{{\text{CNN}}}} {\text{(n) = }}\left[ {\begin{array}{*{20}c} {H_{{\text{L}}} ({\text{t - 1}})} & {H_{{\text{L}}} ({\text{t}})} & {...} & {H_{{\text{L}}} ({\text{t - n}})} \\ {R({\text{t)}}} & {R({\text{t - 1)}}} & {...} & {R({\text{t - n + 1)}}} \\ {R_{{\text{m}}} ({\text{t,n)}}} & {R_{{\text{m}}} ({\text{t - }}1,{\text{n)}}} & {...} & {R_{{\text{m}}} ({\text{t - n + }}1{\text{,n)}}} \\ \end{array} } \right]$$

Based on the Copula analysis, previous items of the *H*_L_ have the highest correlation with its current item, comparing to the daily rainfall and average rainfall. Therefore, the optimized input data set for CNN at time t, denoted as $V_{{{\text{CNN}}}}^{*}$, is presented as,

10 $$V_{{{\text{CNN}}}}^{*} {\text{(n) = }}\left[ {\begin{array}{*{20}c} {H_{{\text{L}}} {\text{(t}} - {1)}} & {H_{{\text{L}}} {\text{(t}} - {2)}} & \cdots & {H_{{\text{L}}} {\text{(t}} - {\text{n)}}} \\ \end{array} } \right]$$

**Figure 1:**
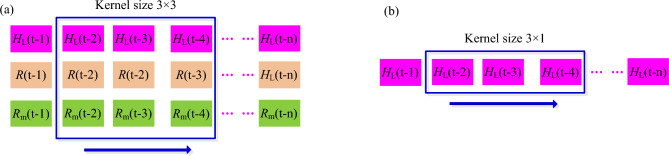
One-dimensional convolutional kernel sliding with different input for the CNN model (**a**) optional input; (**b**) optimized input. The figure shows the one-dimensional convolutional kernel sliding over the inputs with one variable (optimized) and three variables (optional) for the CNN model. The kernel width is set to be 3 for the two different inputs, and the heights for the inputs with three variables and one variable are 3 and 1, respectively.

With the input of Eqs. (9) or (10), the output of the CNN model is one value of the predicted low frequent component at time t, denoted as *H*_Lp(t)_. Figure 1 shows the one-dimensional convolutional kernel sliding over the optional and optimized input. The kernel width is set to be 3 for the two inputs, but the heights for the optional and optimized inputs are 3 and 1, respectively, as show in Fig. 1.

Furthermore, for one dimensional convolution, the k-th convolution is to extract the feature graph *Y*_k_ of the kernel by performing operations on the data *X*_ij_ within the kernel. The convolution operation formula is

11 $$Y_{{\text{k}}} = g\left( {\sum\limits_{{{\text{i}} = 1}}^{{{\text{width}}}} {\sum\limits_{{{\text{j}} = 1}}^{{{\text{height}}}} {X_{{{\text{ij}}}} } } \otimes W_{{{\text{ij}}}} + b_{{{\text{ij}}}} } \right)$$

where, the width and the heigh are determined by the kernel size; $\otimes$ represents convolutional operation; *W*_ij_ is the weight coefficient of the k-th kernel in the layer; *b*_ij_ is the bias coefficient of the k-th kernel in the layer. *g*(*x*) is the RELU activation function with

12 $$g = \max (Y_{{\text{k}}} ,0)$$

And for each time, the training of the CNN is based on the mean square error MSE(*H*_L_), which depend on the difference between the predicted value *H*_Lp_(t)and the actual value* H*_L_(t) at time t, as

13 $${\text{MSE}}(H_{L} {)} = \left( {H_{L} ({\text{t}}) - H_{Lp} ({\text{t}})} \right)^{2}$$

### The LSTM neural network for prediction of *H*_H_

For high frequency component, its changes are quite complex and show significant volatility. According to previous studies, the LSTM model provides good prediction in time series with volatility^62,63^. Therefore, LSTM was adopted to predict the high frequency component. And for the LSTM model, there are one hidden layer and 50 neurons adopted, and the optional input data set, denoted as $V_{{{\text{LSTM}}}}$ with,

14 $$V_{{{\text{LSTM}}}} {\text{(n) = }}\left[ {\begin{array}{*{20}c} {H_{{\text{H}}} ({\text{t}} - 1)} & {H_{{\text{H}}} ({\text{t}} - 2)} & {...} & {H_{{\text{H}}} ({\text{t}} - n)} \\ {R({\text{t}})} & {R({\text{t}} - 1)} & {...} & {R({\text{t}} - {\text{n + 1}})} \\ \end{array} } \right]$$

Based on the Copula analysis, the correlation between the items of the high-frequency component is relatively low comparing to the daily rainfall. Therefore, the optimized input data set $V_{{{\text{LSTM}}}}^{*}$ is supposed to be one dimensional array as,

15 $$V_{{{\text{LSTM}}}}^{*} {\text{(n) = }}\left[ {\begin{array}{*{20}c} {R({\text{t}})} & {R({\text{t}} - {1})} & \ldots & {R({\text{t}} - {\text{n}})} \\ \end{array} } \right]$$

And with the input of Eqs. (14) or (15), the output for the model is the predicted high frequency component at time t, denoted as *H*_Hp_(t). Furthermore, the training of the LSTM is based on the mean square error MSE(*H*_H_) as,

16 $${\text{MSE}}(H_{H} ) = \left( {H_{H} (t) - H_{HP} (t)} \right)^{2}$$

where, *H*_H_(t) are the actual frequency components at time t.

### The ANN deformation prediction model

**Figure 2:**
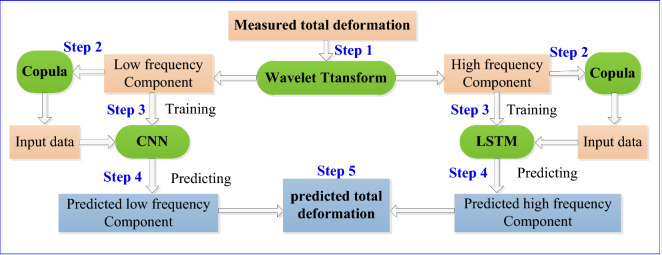
Flowchart of the proposed ANN deformation prediction model. There are 5 steps in the procedure of the ANN model in prediction deformation. Step 1, perform wavelet decomposition, decompose the deformation time series into two components. Step 2, use Copula method to analyze the correlation of the two components with different variables, and determine and optimize the input variables of the ANN model. Step 3, input the obtained data of the input variables into the CNN and LSTM neural networks, and establish CNN and LSTM prediction models by training these neural networks. Step 4, use the CNN and LSTM to predict the two components for the future. Step 5, add the two components to obtain the final deformation prediction values.

Based on the WT method, Copula method, CNN and LSTM neural network, an ANN deformation prediction model for foundation pit was proposed with the flowchart shown in Fig. 2. The on-site measuring deformation time series obtained is denoted as *H*(t−1), *H*(t−2),⋯, *H*(t−n). Based on Fig. 2, the procedure of the ANN model is described as following.

Step 1, perform wavelet decomposition, decompose the time series *H* into two sub-sequences, *H*_L_ and *H*_H_.

Step 2, use Copula method to analyze the correlation of the components *H*_L_ and *H*_H_ with different variables, and determine and optimize the input variables of the ANN model.

Step 3, input the obtained data of the input variables into the CNN and LSTM neural network, and establish CNN and LSTM models by training these neural networks.

Step 4, use the CNN and LSTM neural network to predict low frequency components *H*_Lp_(t) to *H*_Lp_(t + m), and high frequency components *H*_Hp_(t) to *H*_Hp_(t + m), respectively.

Step 5, add the corresponding low-frequency and high-frequency components to obtain the final predicted total deformation. That is,

17 $$\begin{gathered} H_{{\text{P}}} ({\text{t}}) = H_{{{\text{Lp}}}} ({\text{t}}) + H_{{{\text{Hp}}}} ({\text{t}}) \hfill \\ H_{{\text{P}}} ({\text{t + 1}}) = H_{{{\text{Lp}}}} ({\text{t + 1}}) + H_{{{\text{Hp}}}} ({\text{t + 1}}) \hfill \\ ... \hfill \\ H_{{\text{P}}} ({\text{t + m}}) = H_{{{\text{Lp}}}} ({\text{t + m}}) + H_{{{\text{Hp}}}} ({\text{t + m}}) \hfill \\ \end{gathered}$$

where, *H*_p_(t), *H*_p_(t + 1),…, *H*_p_(t + m) are the prediction total deformations for the time t to the time t + m.

**Figure 3:**
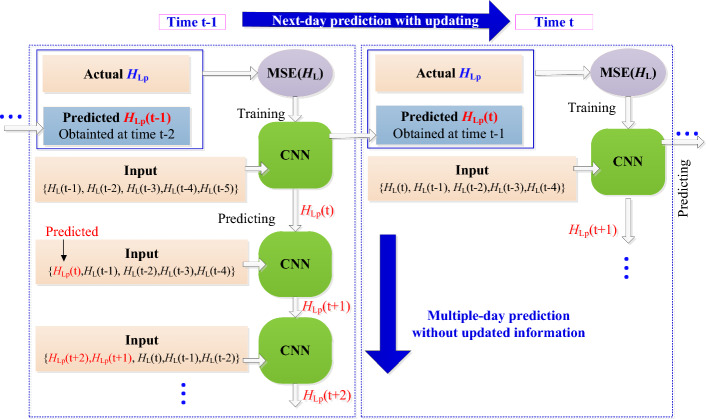
Rolling training and prediction procedure for CNN Neural network. The parameters of the CNN neural network were updated with the rolling training each day, and the next-day predictions are conducted with the updated model, as rolling training and prediction for each day. And for the multi-day prediction is required, the corresponding input data can be replaced by the predicted values but without updating the CNN model.

The CNN neural network model adopts rolling training and prediction method, as shown in Fig. 3. Assuming that a new deformation data *H*(t) is obtained at time t, and thus the low frequency components* H*_L_(t), *H*_L_(t−1)…, *H*_L_ (1) can be obtained by wavelet decomposition. By comparing the predicted low frequency component *H*_Lp_(t) with the corresponding actual value *H*_L_(t), the mean square error MSE(*H*_L_) can be calculated, and the CNN model is then trained and updated. And then the input variables with new obtained data are input into the updated CNN model to predict the next item of the time series, is denoted as next-day prediction. And at each subsequent time, a new deformation data is obtained, the training and prediction process is repeated again as time t, as rolling training and prediction. If multi-day prediction is required, the corresponding input data can be replaced by the predicted values but without updating the CNN model, as the multiple-day prediction at time t−1 shown in Fig. 3.

**Figure 4:**
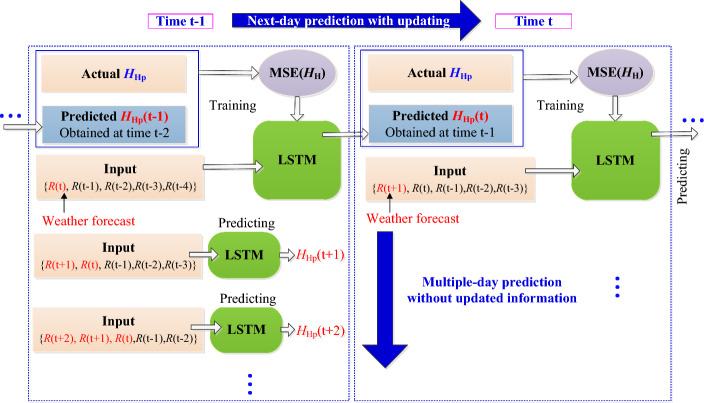
Rolling training and prediction procedure for LSTM neural network. The parameters of the LSTM neural network were updated with the rolling training each day, and the next-day predictions are conducted with the updated model, as rolling training and prediction for each day. And for the multi-day prediction, the corresponding input data can be replaced by the rainfall for weather forecast but without updating the LSTM model.

The LSTM neural network also adopts rolling training and prediction method, as shown in Fig. 4. The LSTM neural network is trained daily according to the MSE(*H*_H_). After each training step, the parameters of the LSTM model are updated accordingly. Then the next day prediction with the updated LSTM model is conducted. For each subsequent moment, a new measurement deformation data will be obtained and the new iterative training is then conducted. If multi-day prediction is required, the corresponding daily rainfall can be obtained from weather forecasting without updating the LSTM model, as the multiple-day prediction at time t−1 shown in Fig. 4. And the predicted values of the low and high frequency components at the corresponding moment are added to obtain the final prediction results of the deformation using Eq. (17).

## Case study

### Project profile

**Figure 5:**
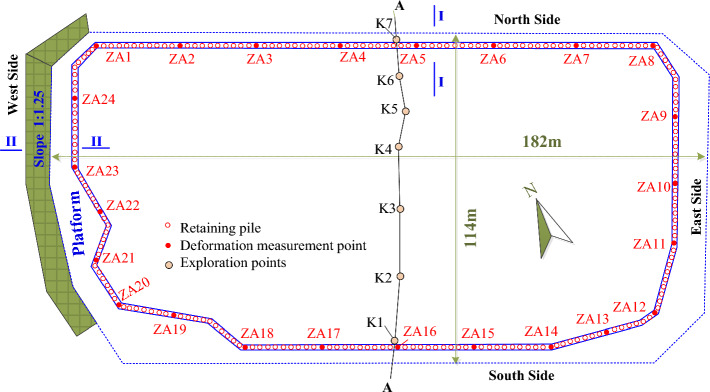
Plan of the deep foundation pit. The plan of a deep foundation pit project in Chengdu City, China is shown in the figure. The excavation depth of the foundation pit is 11.5 m to 14.3 m, and the width and length of the foundation pit plan are 114 m and 182 m respectively.

**Figure 6:**
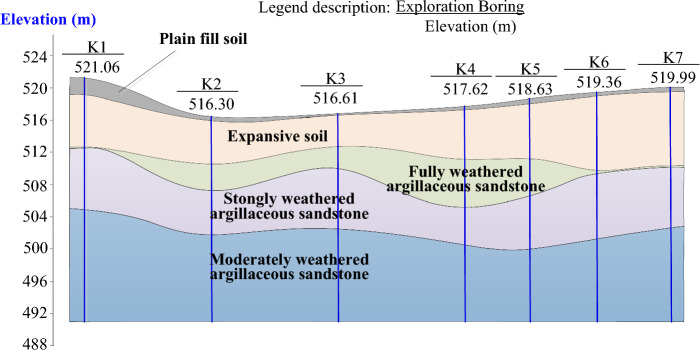
Geological profile at A-A section. There are seven exploratory borings along the A-A section in for the deep foundation pit, and the obtained geological profile is shown in the figure. The surface of the construction site is covered with approximately 0.5 m of plain fill soil; The underlying expansive soil layer has a thickness about 0.7 m to 12.7 m; fully-weathered, strongly weathered and moderately-weathered argillaceous sandstone are distributed below the expansive soil layer.

A deep foundation pit project in Chengdu City, China is shown in Fig. 5. The excavation depth of the foundation pit is 11.5 m to 14.3 m, and the width and length of the foundation pit plan are 114 m and 182 m respectively. There are seven exploratory borings, denoted as K1 to K7, along the A-A section in Fig. 5, and the obtained geological profile is shown in Fig. 6. The surface of the construction site is covered with approximately 0.5 m of plain fill soil; The underlying expansive soil layer has a thickness about 0.7 m to 12.7 m; fully-weathered, strongly weathered and moderately-weathered argillaceous sandstone are distributed below the expansive soil layer. The expansive layer is widely distributed in the construction site. The test results of undisturbed soil samples retrieved from the expansive soil layers show that the mineral composition, Atterberg Limits, and swelling properties of soil samples at different depths are basically the same. However, the water content of shallow expansive layer is significantly higher than that of deep expansive layer, resulting in large differences in both strength and deformation parameters, as shown in Table 1.

**Table 1:** Physical and mechanical properties of samples retrieved form the expansive soil layers.

Clay mineral composition content			Physical parameters and indexes				
Kaolinite	Illite	Montmorillonite	Specific gravity	Plastic limit	Liquid limit	Expansibility	Free swelling ratio
19.7%	66.5%	13.8%	2.7	20.4%	39.6%	76.2 kPa	67%

Mechanical parameters change with depth and water content							
Depth	Water content (%)	Void ratio	Compressive modulus *E*_s_ (MPa)	Cohesion (kPa)	Friction angle *φ*		
1.5–2 m	33.1	0.940	5.0	6.63	5.16^o^		
6.5 ~ 7 m	21.9	0.629	10.6	71.53	28.32^o^		

The expansive soil layers were revealed in the construction site of the foundation pit for the case study in the paper. The X-ray diffraction (XRD) tests was conducted to reveal the mineral components on the expansive soil specimens retrieved from the expansive soil layers. And the laboratory tests were conducted to reveal the Atterberg Limits, swelling properties, water content, compressive and strength parameters for these expansive soil samples. It is found that the mineral composition, Atterberg Limits, and swelling properties of soil samples at different depths are basically the same, but due to the water content of shallow expansive clay layer is significantly higher than that of deep expansive clay layer, the compressive and strength parameters are quite difference. The higher the water content, the lower the strength and the higher the compressibility.

**Figure 7:**
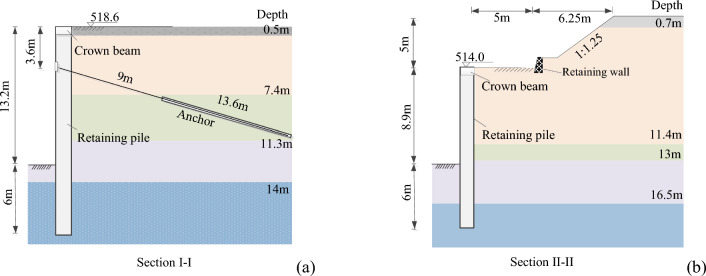
Retaining structures of the deep foundation pit: (**a**) Section I-I; (**b**) Section II-II. Two different retaining schemes were adopted for the foundation pit. The first scheme is the pile anchor support scheme, which is used on the south, north, and east sides of the foundation pit; and the second scheme is slope excavating at the upper part and supporting the lower part by cantilever piles, which is used on the west side. The retaining structures at I-I section adopt the first scheme (Fig. 7a), with an excavation depth of 13.2 m. The retaining piles have a diameter of 1.2 m and length of 19.2 m, the spacing between the piles is 2.0 m, and the embedded depth of the retaining piles is 6.0 m. At the depth of 3.6 m, a row of prestressed anchor cables with a length of 22.6 m is set for the retaining piles. And the soil between the piles is sealed using a reinforcement mat with shotcrete. The retaining structures at II-II section adopt the second scheme (Fig. 7b), with an excavation depth of 13.9 m. The retaining piles have a diameter of 1.2 m and length of 14.9 m respectively, and the spacing between the piles is 2.0 m. The embedded depth of the retaining piles is 6.0 m. The depth of the slope excavation at this section is 5 m with an inclination of 1: 1.25, and a platform with a width of 5.0 m was designed behind the retaining piles. A 1.5 m high rubble concrete retaining wall is constructed at the foot of the excavation slope.

Two different retaining schemes were adopted for the foundation pit, as shown in Fig. 7. The first scheme is the pile anchor support scheme, which is used on the south, north, and east sides of the foundation pit; and the second scheme is slope excavating at the upper part and supporting the lower part by cantilever piles, which is used on the west side. The retaining structures at I-I section adopt the first scheme (Fig. 7a), with an excavation depth of 13.2 m. The retaining piles have a diameter of 1.2 m and length of 19.2 m, the spacing between the piles is 2.0 m, and the embedded depth of the retaining piles is 6.0 m. At the depth of 3.6 m, a row of prestressed anchor cables with a length of 22.6 m is set for the retaining piles. And the soil between the piles is sealed using a reinforcement mat with shotcrete. The retaining structures at II-II section adopt the second scheme (Fig. 7b), with an excavation depth of 13.9 m. The retaining piles have a diameter of 1.2 m and length of 14.9 m respectively, and the spacing between the piles is 2.0 m. The embedded depth of the retaining piles is 6.0 m. The depth of the slope excavation at this section is 5 m with an inclination of 1: 1.25, and a platform with a width of 5.0 m was designed behind the retaining piles. A 1.5 m high rubble concrete retaining wall is constructed at the foot of the excavation slope.

### Monitoring results

**Figure 8:**
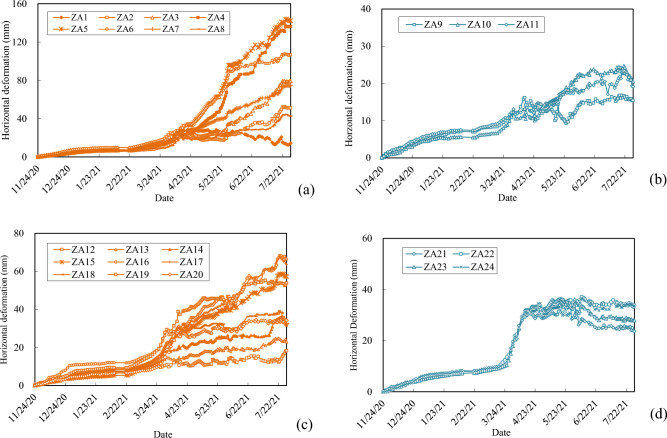
The monitored results of the horizontal deformation on the top of the retaining piles (**a**) north side; (**b**) east side; (**c**) south side; (**d**) west side. The monitoring of the horizontal deformation at the top of retaining piles began on November 24th, 2020, after the construction of the retaining piles. And 24 horizontal deformation measurement points, noted as ZA1 to ZA24, were arranged on the top of the retaining piles. The monitored deformation results for the retaining piles are shown in the figure. And measuring points ZA1 to ZA8 were on the north side (**a**); measuring points ZA9 to ZA11 were on the east side (**b**); ZA12 to ZA20 were on the south side (**c**); and ZA21 to ZA24 were on the west side (**d**).

The construction of retaining piles for the foundation pit were finished before November 24th, 2020. After then, the foundation pit was excavated in layers, and the monitoring of the horizontal deformation at the top of retaining piles began at the same time. As shown in Fig. 4, 24 horizontal deformation measurement points, denoted as ZA1 to ZA24, were arranged on the top of the retaining piles. The monitored horizontal deformations for the different measurement points on the top of the retaining piles are shown in Fig. 8.

**Figure 9:**
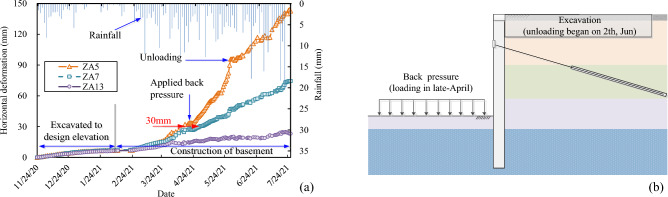
The construction conditions and rainfall during the monitoring period (**a**) the three types deformation curves along with the rainfall and construction information; (**b**) treatment measures for controlling the deformation at section I-I. (**a**) Shows that the whole deformation process curves of the retaining piles for different measuring points can be divided into three types, as stable, less stable and unstable types, respectively. The measuring deformation curves at measuring points ZA13, ZA7 and ZA5 are typically stable, less stable and unstable types of curves respectively. And the construction conditions and rainfall during the deformation monitoring period are also shown in (**a**). (**b**) Shows the treatment measures for controlling the deformation at section I-I. The horizontal deformation of the measurement point ZA5, near section I-I, exceeded 30 mm in mid-April. In order to control the deformation, the back-pressure was applied locally on the north side of the bottom of the foundation pit in late-April. In late-May, the maximum deformation rate at ZA5 reached 7.6 mm/d. In order to further control the deformation and prevent collapse of the foundation pit, partial excavations outside of the retaining piles both on the north and south sides of the foundation pit were carried out on 2th June.

According to the results in Fig. 8, the whole process of these deformation curves of the retaining piles at different measuring points can be divided into three types according to their developing trend, as stable, less stable and unstable types, respectively, and typical deformation curves for the three types are shown in Fig. 9a. Construction conditions and rainfall during the deformation monitoring period are also shown in Fig. 9a. According to the Chinese code (GB 50497-2019)^69^, the early warning values for the cumulative horizontal displacement and displacement rate for the retaining piles of the foundation pit in the study are 30 mm and 3 mm/day, respectively. The rainfall in the region began to increase in late February, and the deformation of ZA5, ZA7, and ZA13 measuring points also significantly increased. As for the stable type deformation curve of ZA13, the deformation gradually stabilized in late March and the final deformation does not exceed the warning value, indicating that the soils and retaining structures near the monitoring point were under stable state. While for the unstable curve of ZA5 and less the stable curve of ZA7, although their deformation rates were less than 3 mm during the period from February to April, their total deformation exceeded 30 mm in mid and late April, respectively, after a period of accumulation, which indication that some zones of the soil strata near the measuring points were under the post-failure stress state and there might be a potential risk of damage to the support structures. At present, the prediction of deformation for foundation is mainly carried out for the stable-typed deformation curves. If the development of less stable and unstable types of the deformation curves can be accurately predicted, it might provide an effective approach for early risk warning for deep foundation pits.

**Figure 10:**
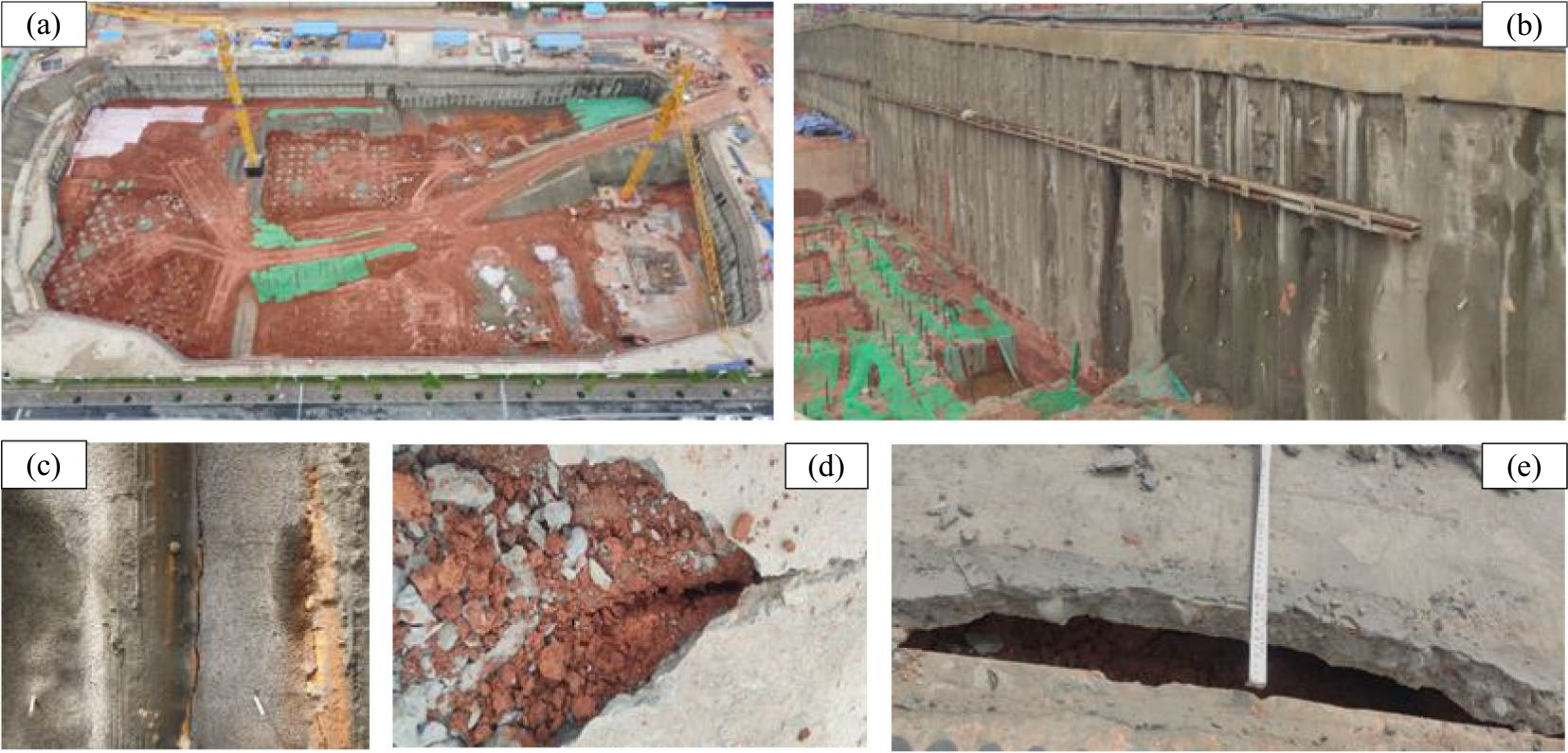
Photographs of the foundation pit (**a**) water seep out at the northeast corner; (**b**) water seep out from the soils between the piles; (**c**) cracks between the piles and soils; (**d**) cracks on the slab and surface of the soil layers; (**e**) cracks between the crow beam and slab. The figure shows photographs of the foundation pit before the back-pressure applying. The long-term rainfall and rainwater infiltration induced water seeped out of the soil between the piles (**a**,**b**); cracks appeared between the piles and the soils (**c**); cracks appeared on the slab and shallow expansive soil layer (**c**), and cracks appeared between the crow beam and slab (**e**).

In March, the rainfall increased gradually, and the horizontal deformation of the measurement point ZA5 exceeded 30 mm in mid-April. As shown in Fig. 9b, in order to control the deformation, the back-pressure was applied locally on the north side of the bottom of the foundation pit in late-April, shown as in Fig. 9b at Section I-I. Figure 10 shows photographs of the foundation pit before the back-pressure applying. The long-term rainfall and rainwater infiltration induced water seeped out of the soil between the piles; cracks appeared between the piles and soils, on the slab and shallow expansive soil layer, and between the crown beam and slab. In late-May, the maximum deformation rate at ZA5 reached 7.6 mm/day. In order to further control the deformation and prevent collapse of the foundation pit, partial excavations outside of the retaining piles both on the north and south sides of the foundation pit were carried out on June 2nd, as illustrated in Fig. 9b with Section I-I, after which the deformation rate slowed down. And at the end of the construction of the basement on mid-June, the horizontal displacement of ZA5, near the middle of the north side, gradually stabilized after reaching 142.1 mm. Due to the unique engineering properties, expansive soil poses a significant risk of instability in foundation pits under rainfall, and reasonable prevention measures need to be adopted as early as possible.

## Results

### The input variables for the ANN deformation prediction model

**Figure 11:**
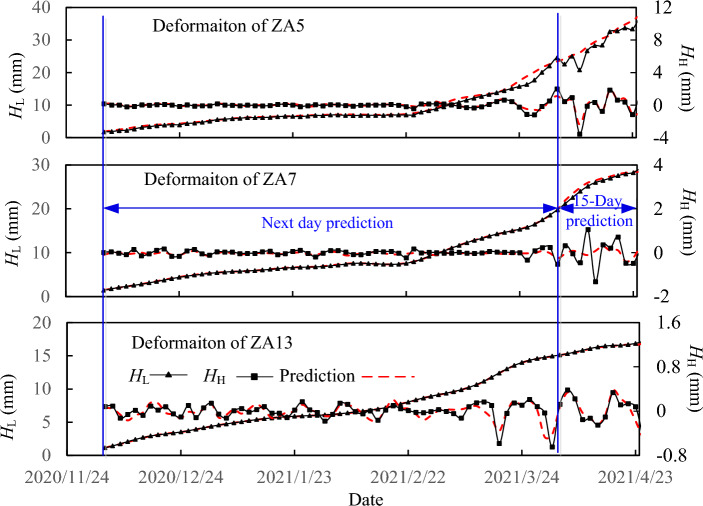
Comparisons of the actual and predicted low and high frequency components for the measuring points ZA5, ZA7 and ZA13. The decomposition results for the deformation curves of ZA5, ZA7 and ZA13 are shown in the figure. The actual decomposed components and the correspondingly predicted components for ZA5, ZA7 and ZA13 are also present in the figure. Assuming that the current moment was April 13th, 2021, and the rolling training and prediction was carried out on December 5th, 2020 to April 13th, 2021. Before April 13th, 2021, the next-day prediction values are present and after April 13th, 2021, the 15-day predication values are present in the figure. The figure shows that both the CNN and LSTM networks have high prediction accuracies.

**Figure 12:**
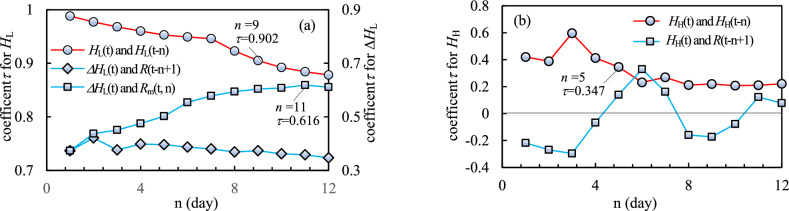
The correlation analysis results (**a**) the Kendall Rank Correlation Coefficient for low frequency components; (**b**) the Kendall Rank Correlation Coefficient for high frequency components. The higher the value of the Kendall Rank Correlation Coefficient, the higher the correlation between the variables. Therefore, the low frequency components within previous 9 days are all highly correlated with the current low frequency component. The previous average rainfall has high to medium correlation with the rate of the current low frequency component, while the daily rainfall has low to medium correlation with the rate of the current low frequency component. The high frequency component at current time t is moderately correlated with the rainfall at current time and within previous 4 days, while is relatively low correlated with the previous high frequency component.

The decomposition results for the deformation curves of ZA5, ZA7 and ZA13 is shown in Fig. 11. The Copula method was programmed using MATLAB. And then, the value of *τ* for both the low frequency component *H*_L_(*t*), and high frequency component *H*_H_(*t*) with different random variables, are calculated, as shown in Fig. 12.

As shown in Fig. 12a, *H*_L_(t) has high correlation with *H*_L_(t−n), and for n less than or equal to 9 days, the values of *τ* are greater than 0.9. The average rainfall has medium correlation with ∆*H*_L_(*t*), with highest value 0.616 with n = 11, while the daily rainfall has low to medium correlation with ∆*H*_L_(*t*) within the past 12 days. The results of the correlation analysis imply that the comprehensive impact of various influence factors, including previous rainfall, is reflected in the development trend of the time series itself. Therefore, the optimized input data for the CNN was supposed to be the values of *H*_L_ within previous 9 days. And as shown in Fig. 12b, *H*_H_(t) has relatively low correlation with *H*_H_(t−n), and *H*_H_(t) is moderately correlated with the rainfall *R* at current time and previous 4 days. Therefore, the short-term daily rainfall can be an optimized selected for the LSTM model to predict *H*_H_.

**Figure 13:**
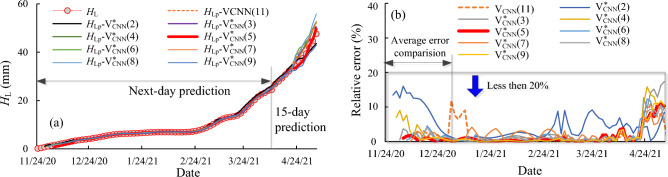
The comparison for the different inputs for the CNN model at the measurement points ZA5 (**a**) the comparison between the actual and predicted low frequency components; (**b**) the relative error for the predictions with different input. Ten different inputs were selected for the CNN model for prediction the deformation at measured point ZA5. When inputting the low frequency components of the previous 5 days yields the highest accuracy in prediction within the year of 2020. And in the entire prediction process, the relative error of all prediction results with different input is less than 20%.

For the CNN model, the initial learning rate is 0.01, and the maximum number of training times in a single day is 500 times. Ten different inputs were selected for the CNN model for ZA5, as in Fig. 13. The different inputs for the CNN model are defined as Eqs. (9) and (10). The average error for ZA5 in the year of 2020, as shown in Fig. 13, with the input $V_{{{\text{CNN}}}}^{*} ({\text{n}})$ and n = 2, 3,.., 9 were 12.83%, 7.92%, 7.63%, 7.25%, 4.32%, 11.20%, 7.89% and 7.64%, and with the input $V_{{{\text{CNN}}}}^{{}} (11)$ was 10.12% respectively. $V_{{{\text{CNN}}}}^{*} (5)$ with the input of the low frequency components of the previous 5 days yields the highest accuracy in prediction within this period. And in the entire prediction process, the relative error of all prediction results with different input is less than 20%, as in Fig. 13.

**Figure 14:**
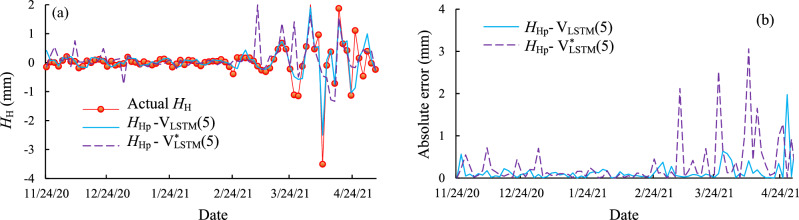
The comparison for the different inputs for the LSTM model at the measurement points ZA5 (**a**) the comparison between the actual and predicted high frequency components; (**b**) the absolute error for the predictions with different input. Two different inputs were selected for the LSTM model for ZA5. The comparison of the absolute error shows that when inputting the daily rainfall at the current and previous 4 days results higher prediction accuracy.

For the LSTM neural network, the initial learning rate is 0.005, and the maximum training number is 500 times per day. Two different inputs were selected for the LSTM model for ZA5, as shown in Fig. 14. The definitions for the input of the LSTM model are defined as Eqs. (14) and (15). The comparison of the absolute error shows that with input $V_{{{\text{LSTM}}}}^{*} (5)$, the daily rainfall at the current and previous 4 days, results higher prediction accuracy.

### Deformation prediction results and early risk warning

**Figure 15:**
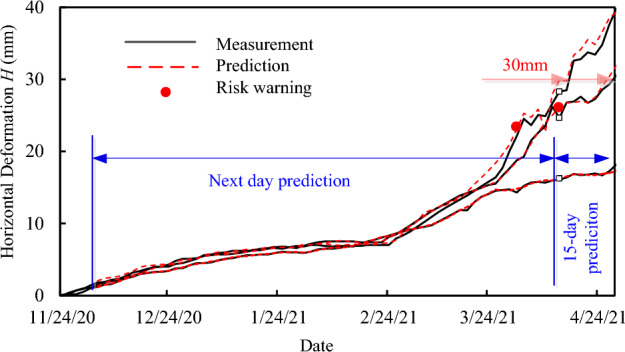
The measured and the predicted total horizontal deformations for ZA5, ZA7 and ZA13 and risk warning promotion. The comparison between the prediction and the measured total horizontal deformation is present in the figure. The input set is supposed to be the previous low frequency components of past five days and the daily rainfall of current and previous 4 days. Assuming that the current moment was April 13th, 2021, and the rolling training and prediction was carried out on December 5th, 2020 to April 13th, 2021. Before April 13th, 2021, the next-day prediction values are present and after April 13th, 2021, the 15-day predication values are present in the figure. The figure shows that the ANN model can predict all the three different types of deformation curves in a high accuracy.

The deformations of ZA5, ZA7 and ZA13 for the deep foundation pit were predicted by the proposed ANN model with the input data set $V_{{{\text{CNN}}}}^{*} (5)$ and $V_{{{\text{LSTM}}}}^{*} (5)$. The data obtained between November 24th and December 4th, 2020, were used as initial training data, with 6 training data sets for both the CNN and LSTM models. The rainfall data in Fig. 9a was used for the deformation prediction of all the three monitoring points of ZA5, ZA7 and ZA13. After the initial model is established, daily rolling training and prediction were carried out. Assuming that the current moment was April 13th, 2021, and the rolling training and prediction was carried out on December 5th, 2020 to April 13th, 2021, during which there are 130 deformation data and 130 daily rainfall data. Before April 13th, 2021, the next-day prediction values are present, and after April 13th, 2021, the 15-day prediction values are presented in Figs. 11 and 15. For each measured point, the initial model was established using only 11 displacement data and 11 rainfall data obtained from November 24th to December 4th, 2020, which can be constructed to 6 training data sets for the ANN model. During the subsequent next-day prediction of rolling training and prediction period from December 5th, 2020 to April 13th, 2021, 130 displacement data and 130 rainfall data were obtained, which combined with the previous data can be constructed to 130 training data sets, and 130 predicting deformation data were obtained correspondingly. On April 14th, 2021, a 15-day prediction was conducted without training updates, during which 15 rainfall data from weather forecasting were input and 15 predicted deformations were obtained. For the 3 measured points, as ZA5, ZA7 and ZA13, the ANN model predicts a total of 435 deformation data, with 145 data for each measurement point, and the comparison between the predicted and the measured data is shown in Figs. 11 and 15. The comparison shows high prediction accuracy with the ANN model in the case of small amount measured data.

The actual deformation value of ZA5 on April 3th, 2021 was 24.53 mm, and the 15-day prediction results of the ANN model on that day shows that 11 days later, on April 14th, the deformation value of ZA5 would exceed the warning value of 30 mm. So, the ANN model promoted risk warning for the first time on April 3th, 2021, which is much earlier than the actual date when the deformation exceeded 30 mm. According to the subsequent measured data, it was found that the deformation value of ZA5 exceeded 30 mm on April 17th, 2021. For monitoring point ZA7, on April 13th, 2021, the ANN model predicted that its deformation would exceed the warning value 30 mm on April 27th, 2021, thus the ANN model prompted the second risk warning. The measured data showed that the deformation of ZA7 exceeded the warning value 30 mm on April 29th, 2021. The proposed ANN model accurately predicts the time when the deformation reaches the warning value, and the time of the risk warning is greatly advanced, which is helpful for preventing major engineering catastrophes.

## Conclusion

Regarding the deformation prediction of deep foundation pits, an ANN deformation prediction model is proposed based on the WT, Copula method, CNN and LSTM neural networks. The deformation predictions of a deep foundation pit were conducted by the proposed ANN model, and the following conclusions were drawn:

1. The proposed ANN deformation prediction model decomposes the deformation of the deep foundation pit into a low frequency component and a high frequency component, and predicts the two components with CNN and LSTM neural network respectively. The CNN neural network was adopted to predict the low frequency component, and the input variables were selected as the previous low frequency components; and the LSTM neural network was adopted to predict the high frequency component, and the input variables were selected as the current and previous daily rainfalls. Only with a few variables, the proposed model can achieve high prediction accuracy.
2. The parameters of the CNN and LSTM neural networks were updated with the rolling training each day, and the subsequent predictions are conducted with the updated parameters, which guarantees the ANN model can reflect the development trend of the deformation and gain a high prediction accuracy for all the different types of deformation curves. The proposed ANN model can accurately predict the time when the deformation reaches the warning value, and the time of the risk warning is greatly advanced, which is helpful for preventing major engineering catastrophes.

## Data Availability

The data used to support the findings of this study are available from the corresponding author upon request.
